# Comparison of best corrected visual acuity estimates between a custom-made digital chart and the ETDRS chart

**DOI:** 10.48101/ujms.v131.13537

**Published:** 2026-02-17

**Authors:** Zhaohua Yu

**Affiliations:** Gullstrand Lab, Ophthalmology, Department of Surgical Sciences, Uppsala University, Uppsala, Sweden

**Keywords:** Visual acuity, digital chart, ETDRS, logMAR, clinical ophthalmology

## Abstract

**Purpose:**

To compare best corrected visual acuity (BCVA) measurements obtained using a digital visual acuity chart with those from the gold-standard Early Treatment Diabetic Retinopathy Study (ETDRS) chart and to assess differences in measurement variability and examination time.

**Methods:**

Altogether 42 subjects (≥ 55 years) were examined using both charts on two separate occasions. BCVA was recorded in logMAR. Examination time was recorded. Subjects were stratified into four visual acuity classes. A nested analysis of variance (ANOVA) was used to analyze systematic differences and variance components.

**Results:**

A statistically significant difference in BCVA between the charts was found, but the 95% confidence interval (CI) for the mean difference (Digital − ETDRS: −0.03 ± 0.04 logMAR) was below the 0.1 logMAR resolution threshold. No significant interaction was observed between chart type and acuity class. The digital chart significantly reduced examination time by an average of 50 sec (95% CI: ±21). Variance was highest between testing occasions compared with that between-subject and for interaction between chart type and subjects.

**Conclusions:**

The digital chart provides clinically equivalent BCVA estimates compared to the ETDRS chart, with shorter examination time. Its use in routine clinical settings is supported.

## Introduction

Visual acuity (VA) is the most widely used clinical measure of visual function and plays a crucial role in the diagnosis, monitoring, and management of ocular diseases (1, 2). It represents the eye’s ability to resolve spatial details and is typically assessed using optotypes presented on standardized charts. VA is quantified as the minimum angle of resolution (MAR), expressed in arc minutes, and often transformed logarithmically into logMAR for precision and statistical analysis (3). Clinically, VA is measured under optimal optical correction, yielding best corrected visual acuity (BCVA) (4, 5).

A variety of VA charts are available, with the ETDRS (Early Treatment Diabetic Retinopathy Study) chart considered the gold standard in clinical research and trials (6, 7). It builds on the design principles by Bailey and Lovie (8), featuring a logarithmic size progression, uniform optotype legibility, consistent inter-letter spacing, and five Sloan letters per line – attributes that minimize bias. Despite its precision, the ETDRS chart is large and less practical in routine clinical settings due to increased space and time requirements (9). Furthermore, the lack of standardized termination rules can lead to variability in results (10, 11).

With technological advancements, digital VA systems have emerged to address these limitations. Systems such as EVA and COMPlog offer programmable optotype presentation and randomization features, showing comparable accuracy to the ETDRS chart (12–14). These systems have demonstrated advantages in usability, flexibility, and patient interaction, with some digital systems achieving shorter examination times (15), although not all (13).

In the present study, a custom-made digital chart, developed at the Gullstrand Laboratory, Uppsala University, was used. It employs a custom visualization algorithm. Initially, the optotypes decrease horizontally from left to right with only one optotype at each logMAR level. Then a single optotype is displayed at a time, with a dial chart for astigmatism correction. This design aims to reduce examination duration while maintaining accuracy comparable to ETDRS measurements (14, 16, 17). This digital system was designed with two main advantages: firstly, patients read the chart horizontally from left to right, consistent with natural reading habits; secondly, astigmatic axis estimation is accelerated using a digital dial chart rather than manual multidirectional testing.

Despite the increasing availability of digital tools, robust validation is essential before clinical implementation. An ideal VA measurement system should offer precision, reproducibility, and resistance to bias from chart design or scoring rules (18–20).

The present study aims to compare BCVA estimates obtained using the custom-made digital chart and the standardized ETDRS chart. Specifically, it investigates whether the two systems yield systematically different acuity estimates across various VA levels and whether the digital system offers reduced examination time without loss of measurement precision.

## Materials and methods

### Subjects

Participants were recruited from the Ophthalmology Clinic at Uppsala University Hospital. Inclusion criteria required subjects to be 55 years or older, have a VA ranging from 1.0 to −0.1 logMAR, and no ocular disease expected to alter vision within 1 month. Only one eye per participant was tested. Participants ≥ 55 years were included, as this reflects the typical demographic of patients attending an ophthalmology clinic.

Informed consent was obtained in writing from all participants, and ethical approval was granted by the Swedish Ethical Review Authority (Dnr 2017/290). This study conformed with the Declaration of Helsinki.

### Charts

Two VA measurement systems were used: the gold standard ETDRS chart and the custom-made digital chart, developed at the Gullstrand Laboratory, Uppsala University. The ETDRS chart (Preisler Instrument AB, Sweden) was placed at a fixed distance of 4 meters in a room with controlled illumination. The digital chart used a digital projector to present optotypes at the same distance and under identical lighting conditions. Both systems utilized Sloan letters.

**Figure 1:**
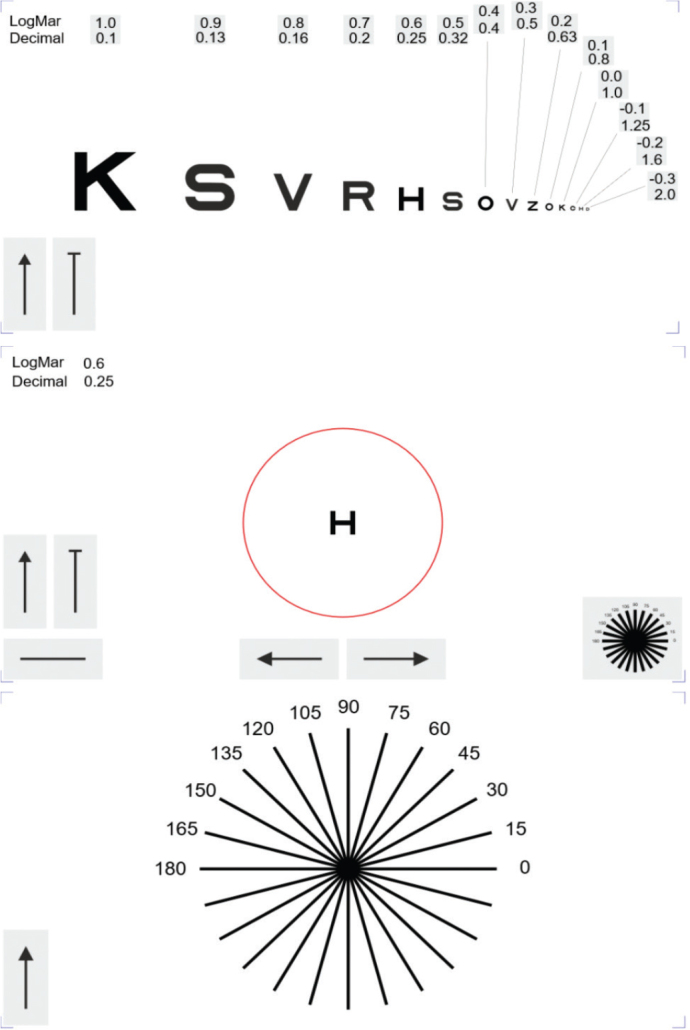
The digital chart. Upper: Optotypes in one line decreasing horizontally from left to right side. Middle: A highlighted optotype within a red circle. Lower: A dial chart for estimating the axis of astigmatism.

The digital system includes a unique algorithm for optotype visualization (Figure 1 Upper) and allows optotypes to be presented individually (Figure 1 Middle). It also incorporates a digital dial chart to facilitate astigmatism axis estimation (Figure 1 Lower). For each logMAR step, only one optotype was presented in the digital chart. While not directly equivalent to the ETDRS full line, this reflects the simplified design intended for clinical use.

### Study design

Subjects were assigned to one of four predefined VA classes ([1.0 0.8], [0.7 0.5], [0.4 0.2], [0.1 −0.1] logMAR) based on the result from the ETDRS chart at the first visit. Each subject underwent testing with both charts on two separate occasions. The order of chart presentation was alternated to control for sequence effects. On each occasion, VA was measured under the same standardized conditions by the same examiner, who was trained in the testing procedure. A different set of optotypes was used for each occasion.

### Testing protocol

Subjects wore a trial frame with one eye occluded during testing. VA was first measured without refractive correction. For the ETDRS chart, subjects read full rows of letters from top to bottom. For the digital chart, subjects read a horizontal line of optotypes from left to right, followed by isolated optotype presentation to refine the acuity threshold.

### Best corrected VA estimation

After initial uncorrected testing, subjective refraction was performed to determine BCVA. Spherical lenses were added incrementally – 1.0 diopter for acuity worse than 0.4 logMAR and 0.5 diopter for acuity 0.4 logMAR or better – until no further improvement was observed.

Astigmatism was corrected using cylindrical lenses. For the ETDRS chart, lenses were manually rotated at angles of 0°, 45°, 90°, and 135° to identify the optimal axis. For the digital chart, the subject used a dial chart interface to indicate the axis, followed by manual fine-tuning. Once astigmatic correction was completed, a final spherical refinement (+0.5 D) was applied.

The BCVA (logMAR), spherical and cylindrical error, axis of astigmatism, chart type, testing order, and total examination time were recorded.

### Definition of resolved vision

For the ETDRS chart, VA was defined as the last full line of five optotypes read correctly. For the digital chart, it was defined as the last individual optotype identified correctly.

### Statistical analysis

The primary outcome variable was BCVA expressed in logMAR. The secondary outcome was examination time in seconds. Explanatory variables included chart type, subject VA class at inclusion, and test occasion.

Agreement between BCVA measurements obtained with the digital chart and the ETDRS chart was assessed using Bland–Altman (B–A) analysis. For each occasion, the difference in BCVA between the two charts (digital chart minus ETDRS chart) was plotted against the mean of the two measurements for each participant. The mean difference represents systematic bias between methods, while the limits of agreement (LoA) were calculated as the mean difference ±1.96 times the standard deviation (SD) of the differences, corresponding to the 95% LoA. Bland–Altman plots were generated separately for the first and second occasions and displayed together to allow visual comparison of agreement across occasions.

The data were analyzed using analysis of variance (ANOVA) to assess: whether there was a systematic difference in VA estimates between the two charts; whether this difference depended on VA class. Prior to analysis, ANOVA assumptions were evaluated by assessing normality and homogeneity of variance among the VA groups. Due to unequal group sizes, the sample size for each VA group in the ANOVA was limited to the size of the smallest group (nine subjects). Participants were randomly selected from larger groups to create equal group sizes.

The model included fixed effects for chart type and VA class and random effects for subjects and test occasions (Appendix 1). Significance level was set at α = 0.05.

## Results

### Subject characteristics

The current study enrolled 42 participants aged from 55 to 93 (Median = 70), with an approximately equal gender distribution (19 women/23 men) (Table 1). Participants were recruited primarily from the Ophthalmology Clinic at Uppsala University Hospital. Most had mild or no refractive error. Subjects with macular degeneration were mainly in lower acuity classes. All completed both test sessions without dropout.

**Table 1:** Sujbect demographics.

Visual acuity classes (logMAR)	Number of subjects (*n*)	Median age (range) (years)	Gender (women/men) (*n*)	Median days between occasions (range) (days)
[1.0 0.8]	10	75.5 (56–93)	5/5	4 (1–18)
[0.7 0.5]	9	71 (55–89)	3/6	1 (1–21)
[0.4 0.2]	12	53.5 (61–85)	5/7	4 (1–28)
[0.1 −0.1]	11	62 (55–74)	6/5	2 (1–17)
Total	42	70 (55–93)	19/23	3 (1–28)

### Test–retest variability for the digital and ETDRS charts

The mean difference in BCVA between the two visits for the digital chart was −0.01 ± 0.04 logMAR (95% confidence interval [CI]), with a test–retest variability (TRV) (SD of differences) of 0.12 logMAR. For the ETDRS chart, the mean difference was −0.02 ± 0.03 logMAR (95% CI), with a TRV of 0.09 logMAR. The digital and ETDRS charts showed comparable TRV in BCVA measurements.

BCVA difference between the digital chart and the ETDRS chart as a function of VA class

**Figure 2:**
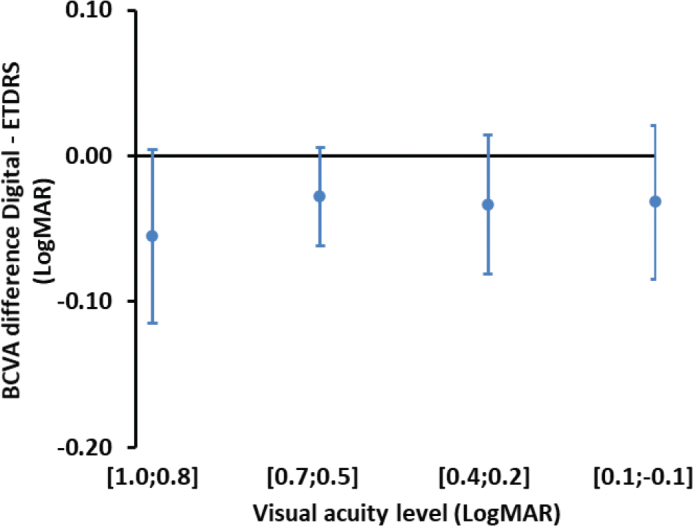
Visual acuity difference between the digital system and the ETDRS chart at different visual acuity classes.

The 95% confidence interval for the mean difference of BCVA between the digital chart and the ETDRS chart (Digital – ETDRS) was estimated −0.03 ± 0.04 logMAR. Figure 2 shows 95% CI for the mean difference at different VA classes.

### Bland–Altman plots between the digital chart and the ETDRS chart

**Figure 3:**
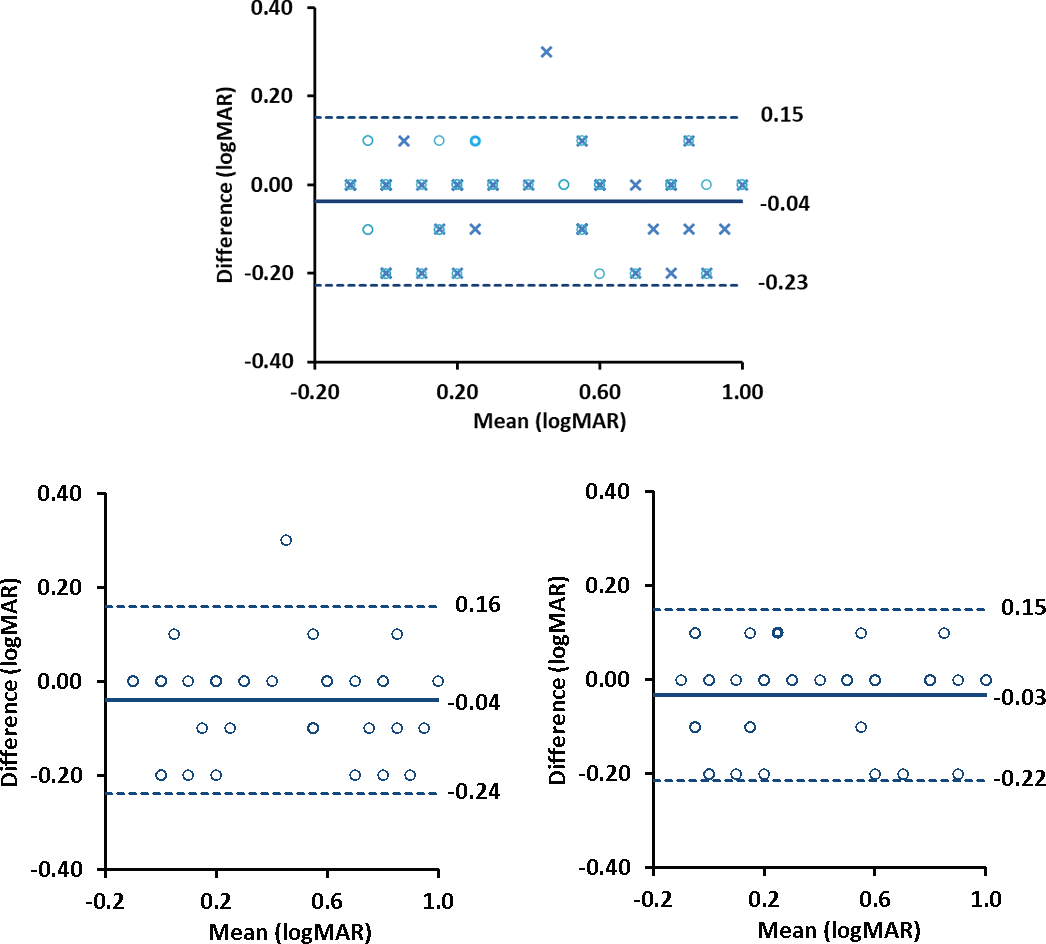
Bland–Altman plots between BCVA measurements obtained with the digital chart and the ETDRS chart (Left: first test occasion; Right: second test occasion). Each point represents the difference between the two charts plotted against the mean for an individual participant. The solid line represents the mean difference (bias), and the dotted lines represent the 95% limits of agreement (mean difference ± 1.96 SD).

Bland–Altman plots demonstrated a high level of agreement in estimated BCVA between the two charts on both occasions (Figure 3).

### Estimates of variance components for VA estimates

ANOVA (Appendix 1) showed a significant difference in VA estimates between the two charts. However, since the difference between the two charts (−0.03 ± 0.04 logMAR) was within a minimum resolution of 0.1 logMAR, this small difference can be considered negligible. There was no significant difference between the two charts depending on VA class.

Estimated variance components for random factors in the model (Appendix 1, Eq. 1) were shown in Table 2.

**Table 2:** Estimate of variance components.

Source	Variance component (logMAR^2^)
Subjects	0.0030
Charts × subjects	4.4724e-18
Occasions	0.0056

The highest variation was observed among occasions, followed by subjects (Table 2).

### Examination time difference between the digital chart and the ETDRS chart

**Figure 4:**
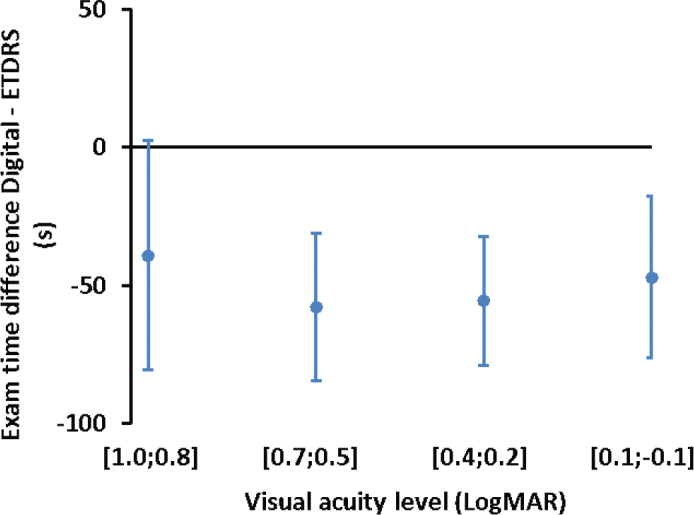
Examination time difference between the digital system and the ETDRS chart at different visual acuity classes.

The 95% CI for the mean difference of examination time (Digital – ETDRS) was estimated as −50 ± 21 s. Figure 4 shows the examination time difference at the different visual acuity classes.

## Discussion

The primary aim of this study was to evaluate whether a custom-made digital VA system could produce BCVA measurements comparable to the gold standard ETDRS chart and whether it could do so in a shorter time. Our results demonstrate that although a statistically significant difference in BCVA estimates was found between the two charts, this difference was smaller than 0.1 logMAR – the smallest detectable step on the ETDRS scale e-indicating that it is clinically negligible.

The digital and ETDRS charts demonstrated similar TRV, reflecting consistent and reliable performance across repeated measurements. The minimal mean differences between visits (−0.01 and −0.02 logMAR) and acceptable SDs of differences (0.12 and 0.09 logMAR) reinforce the reliability of both systems. These results suggest that the digital chart is a viable alternative to the ETDRS chart.

Furthermore, no significant interaction was found between chart type and VA class, indicating that the digital system performed consistently across a range of VA levels. This is particularly important for clinical implementation, as it supports the applicability of the digital chart across different patient populations, including those with mild to moderate vision impairment.

The examination time was significantly shorter using the digital system compared to the ETDRS chart. On average, the digital chart reduced testing time by approximately 1 min. This reduction can be attributed to several design features of the digital chart, such as presenting a single optotype at a time and utilizing an integrated dial chart for astigmatism estimation, thereby streamlining the testing process.

The design of a VA chart directly affects both the accuracy of the measurements and the efficiency of the examination process. One of the most notable differences between the ETDRS chart and the digital system is the number of optotypes presented per VA level. The ETDRS chart displays five optotypes per line, requiring the examinee to read and correctly identify multiple letters to confirm visual performance at that level. In contrast, the digital system displays only one optotype at a time, which simplifies the testing task and may reduce cognitive load for the patient.

While a single-optotype presentation could theoretically lower task difficulty or increase variability, our findings show that this design does not compromise the reliability of the measurement. The minimal difference in BCVA between the two charts and the consistent performance across acuity classes support the idea that fewer optotypes per level do not reduce precision. These findings align with previous digital chart studies where fewer letters per line have also not resulted in compromised accuracy (14, 16).

The built-in dial chart for determining astigmatism axis allows for more intuitive and faster correction compared to the traditional method of rotating cylindrical lenses at fixed angles. This feature contributes to the overall efficiency of the digital system without compromising refractive accuracy.

The findings of this study are consistent with a growing body of literature supporting the validity and efficiency of digital VA systems. For instance, the electronic visual acuity (EVA) system has been shown to yield results in strong agreement with the ETDRS chart across a variety of patient populations (14). Our results align with these findings, showing negligible differences between digital and standard chart measurements.

Similarly, systems like COMPlog have demonstrated equivalent performance to ETDRS in both adults and children (13), with the added benefit of customizable testing parameters. As with the digital system, COMPlog also provides the option for single-optotype presentation. Rosser et al. (16) emphasized the potential of digital charts to reduce TRV, an issue that remains a concern with traditional methods. Our study supports this by showing consistent BCVA results across repeated measures.

In contrast to some earlier digital systems where testing time was equal to or longer than ETDRS (13), our study confirms that the digital chart used in current study reduces testing time without degrading accuracy – an important advantage in clinical workflows where time efficiency is critical.

Room illumination and testing distances were strictly controlled, and subjective refraction was performed before each measurement to ensure accurate BCVA estimation. The inclusion of older adults and individuals with common ocular conditions such as macular degeneration and diabetic retinopathy makes the study population representative of typical ophthalmic patients.

A limitation of this study was the use of the same ETDRS chart for repeated testing, which may introduce memorization bias. Additionally, the lack of crowding in the digital chart design should be considered a limitation, since crowded acuity can better reflect functional vision. However, it may not be critical if the digital chart aims for general routine examination.

However, while the digital chart shows promise in routine clinical work, its applicability in pediatric populations or in non-Sloan optotype systems remains untested. Furthermore, although examination time was significantly shorter using the digital chart, patient-reported experience and preferences were not assessed. Incorporating user feedback in future studies could provide valuable insight into the chart’s acceptability and usability.

## Conclusion

This study demonstrates that the custom-made digital chart provides BCVA estimates that are clinically comparable to those obtained with the gold standard ETDRS chart. Although a statistically significant difference was observed, it remained within the clinically negligible threshold of 0.1 logMAR. The digital chart also significantly reduced examination time, likely due to its streamlined interface, single-optotype presentation, and integrated astigmatism axis estimation. These findings support the use of the digital chart as a reliable and time-efficient alternative for VA assessment in clinical settings.

## Declarations

### Availability of data and material

The datasets used and analyzed in the current study are available from the corresponding author on reasonable request.

## Appendices

### Appendix 1 Analysis Model

A visual acuity, *x_ijkl_*, is equal to the population mean, μ, and a term for the fixed factor chart type, *α_i_* (*i* = 1,2), a term for the fixed factor ETDRS visual acuity level at inclusion, *β_j_* (*j* = 1,2,3,4), and a term for random variation between subjects, *C_k_*_(_*_j_*_)_ (*k* = 1,2,…nj), a term for interaction between chart type and ETDRS visual acuity level at inclusion, *αβ_ij_*, a term for interaction between chart type and subjects, *αC_ik_*_(_*_j_*_)_, and a term for random variation between occasions defined as a measurement error, ε*l*_(_*_ijk_*_)_ (l = 1,2) (Eq. 1).

$$\chi_{ijkl}=+\alpha_{i}+\beta_{j}+C_{k\left(j\right)}+\alpha \beta_{ij}+\alpha C_{ik\left(j\right)}+\varepsilon_{l\left(ijk\right)}$$
Eq.1

The outcome of the measured BCVA will be analyzed with analysis of variance according to Eq. 1 resulting in Table A1.

**Table A1:** Analysis of variance for the estimates of the measured refracted visual acuity.

Source	Degree of freedom	Mean square	Expected mean square
Charts	1	MS_1_	$\sigma_{\varepsilon }^{2}+n\sigma_{\alpha C}^{2}+bcn\kappa_{\beta }^{2}$
Visual acuity levels	3	MS_2_	$\sigma_{\varepsilon }^{2}+an\sigma_{C}^{2}+acn\kappa_{\alpha }^{2}$
Subjects	38	MS_3_	$\sigma_{\varepsilon }^{2}+an\sigma_{C}^{2}$
Charts vs. Visual acuity levels	3	MS_4_	$\sigma_{\varepsilon }^{2}+n\sigma_{\alpha C}^{2}+cn\kappa_{\alpha \beta }^{2}$
Charts vs. Subjects	38	MS_5_	$\sigma_{\varepsilon }^{2}+n\sigma_{\alpha C}^{2}$
Occasions	84	MS_6_	$\sigma_{\varepsilon }^{2}$

a = number of charts. b = number of visual acuity levels. c = number of subjects in each visual acuity level used in analysis of variance. *n* = number of occasions. δ^2^ means the expected mean square for the random factors. Κ^2^ means the expected mean square for the fixed factors.

The analysis of variance allows to test the zero hypotheses:

1) H0: There is no systematic difference between two charts
  Test statistic = MS_1_/MS_5_.
2) H0: There is no systematic difference between the charts depending on visual acuity level
  Test statistic = MS_4_/MS_5_.
